# The dependence of Ig class-switching on the nuclear export sequence of AID likely reflects interaction with factors additional to Crm1 exportin

**DOI:** 10.1002/eji.201041011

**Published:** 2010-11-19

**Authors:** Julia I Ellyard, Amelie S Benk, Benjamin Taylor, Cristina Rada, Michael S Neuberger

**Affiliations:** Medical Research Council Laboratory of Molecular BiologyHills Road, Cambridge, UK

**Keywords:** Antibody diversification, DNA deamination, Hyper-IgM

## Abstract

Activation-induced deaminase (AID) is a B lymphocyte-specific DNA deaminase that triggers Ig class-switch recombination (CSR) and somatic hypermutation. It shuttles between cytoplasm and nucleus, containing a nuclear export sequence (NES) at its carboxyterminus. Intriguingly, the precise nature of this NES is critical to AID's function in CSR, though not in somatic hypermutation. Many alterations to the NES, while preserving its nuclear export function, destroy CSR ability. We have previously speculated that AID's ability to potentiate CSR may critically depend on the affinity of interaction between its NES and Crm1 exportin. Here, however, by comparing multiple AID NES mutants, we find that – beyond a requirement for threshold Crm1 binding – there is little correlation between CSR and Crm1 binding affinity. The results suggest that CSR, as well as the stabilisation of AID, depend on an interaction between the AID C-terminal decapeptide and factor(s) additional to Crm1.

## Introduction

Although it functions in the nucleus to deaminate deoxycytidine residues within the immunoglobulin locus, activation-induced deaminase (AID) is largely located in the cytoplasm but shuttles between nucleus and cytoplasm 1, 2. Nuclear import depends on a variety of basic amino acid residues scattered over the amino-terminal and central portions of AID: these probably form a conformational nuclear localisation sequence 3–6. Export is mediated by a decapeptide nuclear export sequence (NES) located at the very carboxyterminus of the AID polypeptide, with the sequence of this NES conforming well to the consensus established for substrates of the Crm1-dependent export machinery. In keeping with this, the nuclear export of AID is sensitive to the drug leptomycin B, which binds to a conserved cysteine residue in Crm1 7–9.

The lack of switched Ig isotypes found in patients suffering from hyper-IgM syndrome is in many cases due to mutations in the gene encoding AID. Some hyper-IgM patients carry mutations that destroy the catalytic activity of AID with a resulting loss of both Ig class-switch recombination (CSR) and somatic hypermutation. However, in others, the substitutions do not destroy AID's DNA deaminase catalytic activity but rather are missense or truncating mutations affecting the carboxyterminal end of AID. Intriguingly, many of these carboxyterminal mutations, though destroying AID's ability to potentiate CSR, do not preclude its acting in IgV gene somatic mutation 3, 4, 10.

Given that the carboxyterminal AID mutations in hyper-IgM patients are located either within or adjacent to AID's NES, the question obviously arose as to whether loss of CSR was a direct consequence of NES inactivation. A functional NES does indeed seem to be a prerequisite for CSR. All of many point mutations that have been created in the AID NES, which impair its function in nuclear export, have also been found to inhibit its activity in triggering CSR 4, 10–12. This inhibition of CSR occurs (in those cases where it has been tested) without jeopardising somatic hypermutation.

However, provision of nuclear export activity is not the only way in which the AID carboxyterminal region contributes to CSR. Comparison of a family of AID mutants carrying different NES sequences revealed that while a functional NES was essential for CSR, many NES that conferred nuclear export were unable to potentiate CSR 12. Thus, nuclear export activity is a necessary but not sufficient feature of the AID carboxyterminal decapeptide for CSR.

We previously speculated that it might be the affinity of the interaction between the AID NES and Crm1 exportin that is important in determining AID's efficacy in CSR envisioning, for example, that switching might be critically dependent on the rate of AID shuttling or on its residence time at the nuclear pore 12. Here, using AID chimaeras with different NES, we have investigated whether there is a correlation between the affinity for Crm1 and an ability to potentiate CSR.

## Results and discussion

### The AID NES binds Crm1

**Figure 1:**
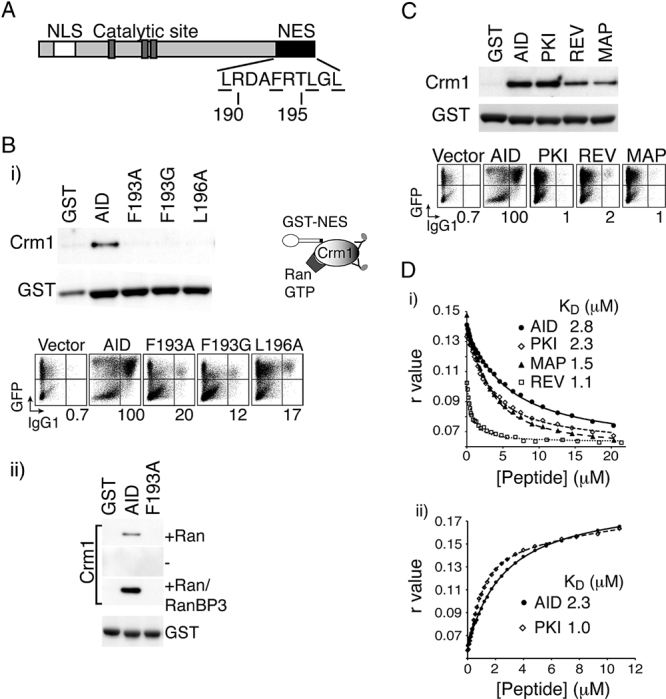
Heterologous NES bind Crm1 with similar affinity to the AID NES but do not facilitate CSR. (A) Schematic diagram of AID highlighting the NES, NLS and Zn-coordination motif. The NES, like other Crm1-dependent NES, is characterised by four hydrophobic residues (underlined) spaced ϕXXXϕXXϕXϕ. (B) Binding of Crm1 to immobilised GST fusion proteins containing the WT or mutant AID NES as monitored by pull-down assays; bound Crm1 was detected by Western blotting. In panel (i) the assays were performed in the presence of GTP-loaded RanQ69L whereas in panel (ii) the incubations contained Crm1 alone, Crm1+Ran or Crm1+Ran+RanBP3 as indicated. Samples were normalised for GST expression. Beneath the gels showing the results of the pull-down assays, flow cytometry profiles reveal the ability of the various AID proteins to facilitate CSR following their expression with an (AID+GFP)-transducing retrovirus in AID^−/−^ B cells. After culture in the presence of LPS+IL4, switching to IgG1 (*x*-axis) was monitored by flow cytometry with GFP fluorescence (*y*-axis) providing a marker of retrovirally infected cells. Representative plots are presented with the numbers giving the percentage (mean of at least two independent experiments) of transfected (GFP^+^) cells that have switched to IgG1 following infection with the mutant AID relative to that obtained with WT human AID. (C) Upper panels: Binding of Crm1, in the presence of GTP-loaded RanQ69L, to immobilised GST fusion proteins containing heterologous NES (MAP, MAP kinase kinase; PKI, protein kinase inhibitor alpha; REV, HIV-1 Rev protein). Lower panels: the switching potentiated by these same NES when fused to AID[ΔNES] is depicted as in panel B. (D) Binding of heterologous NES peptides to Crm1/RanGTP was measured by (i) competitive inhibition fluorescence anisotropy against fluorescein-labelled AID NES peptide and (ii) direct binding fluorescence anisotropy. *K*_D_ values were calculated from triplicate experiments in (i), duplicates in (ii).

We first tested whether the NES of AID does indeed bind Crm1 by asking whether a GST fusion protein containing the AID NES at its carboxyterminus could bind recombinant Crm1 *in vitro*. Binding was observed and was dependent on the presence of Ran-GTP (Fig. 1A and B). It was also enhanced by the addition of the widely expressed Ran-binding protein Ran-BP3, which has been shown to facilitate the formation of Crm1-RanGTP-NES-cargo complexes in other systems, acting as a positive regulator of nuclear export 13. Mutations of the hydrophobic AID NES residues F193 and L196, which have previously been shown to impair CSR and inhibit nuclear export 12 also destroyed Crm1 binding (Fig. 1B).

### Heterologous NES bind Crm1 but yield no CSR

Previous results revealed that replacement of the AID NES by the NES of protein kinase inhibitor alpha (PKI), MAP kinase kinase (MAP) or HIV-1 Rev allows nuclear export activity to be retained but do not allow retention of CSR activity 12. Pull-down assays demonstrate that these heterologous NES do indeed confer Crm1 binding *in vitro* assays although expression of these AID-NES chimaeric proteins in retrovirally transduced B cells from AID^−/−^ mice confirmed that these heterologous NES do not allow CSR (Fig. 1C).

### AID and heterologous NES bind Crm1 with similar, moderate affinity

To test whether the difference in the ability of the AID and the PKI, MAP and Rev NES to potentiate switching could be due to a difference in their affinity for Crm1, we measured the affinity of the different NES for Crm1 using direct binding assays with fluorescein-labelled peptides (monitoring binding by fluorescence anisotropy) as well as binding inhibition assays. The results reveal that all four NES bind Crm1 with reasonably similar affinities (*K*_D_s in the range 1–3 μM) although the PKI, MAP and Rev NES showed very slightly higher affinities than the AID NES (Fig. 1D).

### Mutated export-proficient NES reveal no correlation between Crm1 affinity and CSR

**Figure 2:**
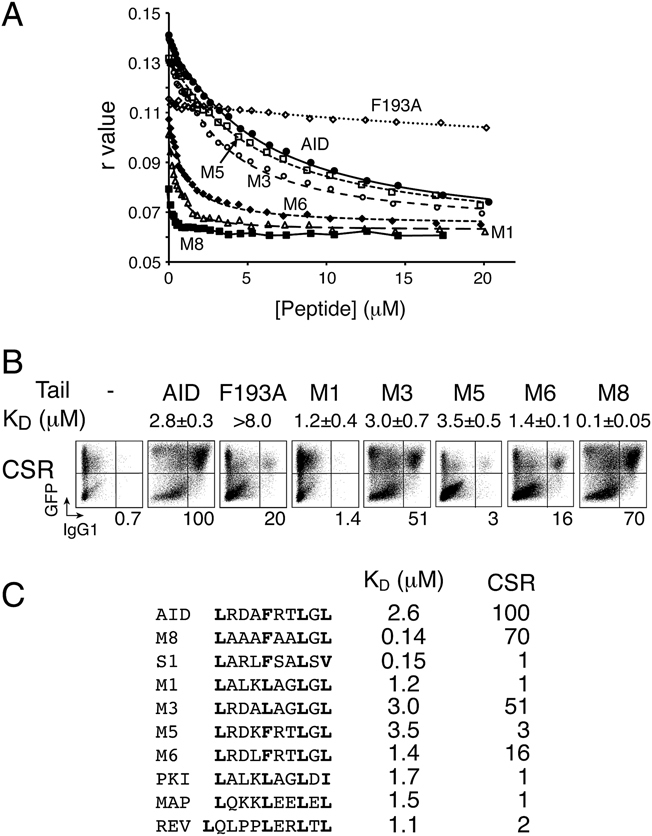
Comparison of Crm1 binding and class-switching by a family of AID NES mutants. (A) Binding of mutant AID NES peptides to Crm1/RanGTP was measured by competitive inhibition fluorescence anisotropy against fluorescein-labelled AID NES peptide and used to calculate *K*_D_ values from three replicates±SEM. (B) The ability of AID proteins containing mutated NES to facilitate CSR in B cells was monitored and displayed as in Fig. 1B. (C) Comparison of the NES sequences used in this work. The sequences of the different NES peptides are indicated along with their affinities for Crm1 (averaging the results obtained from direct binding and binding inhibition assays where relevant) as well as the efficacy with which the relevant AID-NES chimaera directs CSR, expressed as a percentage of that achieved with wild type AID.

We could not exclude the formal possibility that the albeit rather small increase in Crm1 binding affinity displayed by the PKI, MAP and Rev NES as compared to the AID NES might explain the inability of these heterologous NES to potentiate CSR. We, therefore, extended our analysis to set of mutant AID NES, which carry substitutions in the hydrophilic interstitial amino acids of the NES, some of which allow switching (M3 and M8) and others of which do not (M1 and M5), with M6 giving a partial reduction. While all these NES bound Crm1, there was no correlation between the strength of Crm1 affinity and the efficiency of CSR (Fig. 2). Thus, for example, M1, M3 and M8 have an affinity for Crm1 in the rank order M8>M1>M3 and whereas M8 and M3 both give CSR, M1gives none.

**Figure 3:**
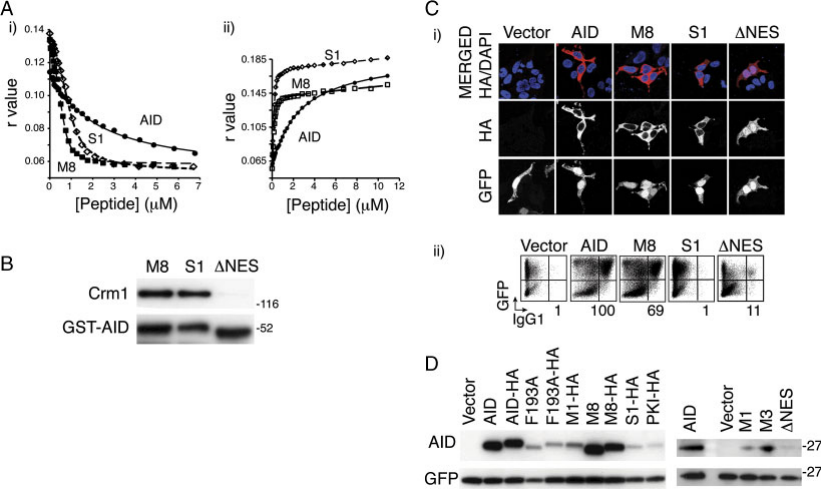
The M8 and S1 NES both bind Crm1 with high affinity but differ with respect to their effects on CSR and AID stabilisation. (A) Binding of M8 and S1 NES peptides to Crm1/RanGTP was measured both by (i) competitive inhibition fluorescence anisotropy with unlabelled peptides (competing with fluoresceinated AID NES peptide) and (ii) direct binding fluorescence anisotropy with fluorescein-labelled peptides. *K*_D_ values were calculated from at least duplicate measurements. (B) Binding of Crm1 to immobilised fusion proteins of GST to full-length AID that comprises either the M8, S1 or no NES in the presence of GTP-loaded RanQ69L. (C) Both S1 and M8 NES mediate nuclear export, whereas only M8 allows CSR. (i) HA-tagged AID WT and mutant proteins were transfected into 293T cells using pMx-GFP vectors. Transfected cells were identified by their GFP fluorescence with the HA-tagged AID proteins localised by staining with anti-HA antibody, counterstaining with DAPI. (ii) Comparison of the ability of the same HA-tagged AID[M8/S1-NES] proteins to facilitate CSR in B cells was monitored as in Fig. 1B. (D) Abundance of mutant AID. Abundance was monitored by Western blotting of extracts of AID^−/−^ B cells that had been cultured for 3 days in the presence of LPS+IL4 following retroviral transduction with the relevant AID-expressing pMx-GFP construct. Blots were reprobed with anti-GFP antibodies to control for loading and infection. The blots are representative of at least two experiments.

In fact, the affinity of M8 for Crm1 (*K*_D_ 90 nM by binding inhibition) is considerably higher than that of all the natural NES tested in this work (*K*_D_ between 1 and 2.8 μM) and falls within the supraphysiological range of that described for an artificial NES peptide that has been isolated by peptide phage display 14. Thus, both M8 and S1 peptides give sub-micromolar binding to Crm1 in direct binding assays (*K*_D_s of 190 and 150 nM for M8 and S1 respectively; Fig. 3A) and both peptides mediate good binding to Crm1 when fused to the C-terminus of a GST-AID[ΔNES] chimaera, (Fig. 3B). Both, when assayed in place of the WT NES in the context of HA-tagged AID, also mediate efficient nuclear export (Fig. 3C). However, whereas HA-tagged AID[M8-NES] gives good CSR, no switching was observed with the HA-tagged AID[S1-NES] chimaera (Fig. 3C). Thus, even within supraphysiologically high affinity range, one NES gives good CSR whereas another does not.

### CSR correlates with AID stabilisation

We previously noted that those NES, which potentiated CSR, also yielded an AID polypeptide that exhibited greater stability in transfected cells and was expressed in greater abundance 12. The same correlation between CSR and AID abundance extends to the new mutants made in this work. Thus, for example, the S1 and M8 NES both give high affinity interaction with Crm1 but the M8 NES potentiates CSR whereas the S1 NES does not. Western blot analysis reveals that the AID[M8-NES] chimaera accumulates to considerably higher abundance in transduced B cells than the AID[S1-NES] (Fig. 3D).

### Concluding remarks

The NES of AID is essential for CSR but must act by more than just providing a signal for nuclear export. Some NES allow nuclear export, CSR and AID stabilisation whereas others just mediate nuclear export without facilitating AID stabilisation and switching. We previously speculated that the distinction between switch-potentiating and switch-nonpotentiating NES might lie in a differential affinity for Crm1 exportin. The reasoning was that such differences might underpin differences in the kinetics of nuclear/cytoplasmic shuttling (thereby affecting AID turnover/abundance and therefore CSR) or that they might lead to differences in the residence time of AID at the nuclear pore, where CSR might take place. Here, however, by measuring the binding affinity of different export-competent NES for Crm1, we find no evidence of a correlation between Crm1 affinity and the ability to potentiate CSR.

Where then does that leave us with respect to mechanism by which the AID NES functions in CSR? Although we cannot formally exclude the possibility that switch-proficient and switch-incompetent NES differ in their affinity of interaction with Crm1 under some unidentified aspect of *in vivo* conditions that are not recapitulated in our *in vitro* assays, it would seem more likely that AID stabilisation and CSR require an interaction between the C-terminal amino acids of AID and some as yet unidentified cellular factor apart from Crm1. Such an interaction might lead to AID stabilisation and thereby allow effective CSR. Intriguingly, Patenaude *et al*. 6 have also inferred the existence of an as yet unidentified partner that interacts with the AID C-terminus but in that case implicated in AID's cytoplasmic retention. It may be that the identification of physiologically relevant factors interacting with the AID C-terminus will be assisted by the delineation here of those NES sequences that do facilitate CSR and those that do not.

## Materials and methods

### Protein expression and purification

Recombinant humanRanQ69L, RanBP3 and *T7*•*Tagged-*Crm1 were purified as His6 fusion proteins from extracts of *E. coli* Rosetta (DE3) cells transformed with the relevant human cDNAs cloned in pET-28a(+) (Novagen) by binding onto Ni-NTA-Sepharose and elution with 250 mM imidazole. pGEX-hCrm1 was a gift from Dr. Yu-Min Chook (UT Southwestern Medical Center, Dallas) and GST-TEV-hCrm1 was purified as previously described 15. GST-NES fusion proteins were expressed in *E. coli* Rosetta (DE3) cells transformed with pGEX-4T-1 (GE Healthcare) containing the relevant NES sequences. GST fused to full-length human AID was generated by PCR-mutagenesis of pOPTG and expressed in BL21 (DE3) *E. coli*.

### GST pull-down assay

GST-NES and GST-full-length AID fusion proteins in 50 mM-sodium phosphate pH 7.5, 300 mM-NaCl, 5 mM 2-ME were bound to glutathione-Sepharose and, after blocking with 5% BSA and washing, resuspended in 20 mM-HEPES pH 7.4, 300 mM-NaCl, 2 mM-MgCl_2_, 0.1% Tween-20, 0.1% BSA, 28 mM 2-ME containing 0.5 μM *T7*•*Tagged-*Crm1 and 1 μM RanQ69L that had been pre-charged with GTP as described 16. Following SDS/PAGE, bound Crm1 was detected by Western blotting with HRP-conjugated *T7*•*Tag*® (Novagen).

### Fluorescence anisotropy

Peptides were synthesised by Peptide Protein Research (Funtley, Fareham, UK), Designer Bioscience (Cambridge, UK) or Pepnome (Shuzhou City, China). Peptide sequences are as shown in Fig. 2C with an N-terminal tryptophan added to allow determination of concentration. Fluorescein-labelled peptides included an N-terminal glycine residue to facilitate fluorescein conjugation. In direct binding assays, Crm1/RanQ69L-GTP protein mix (1:1 molar ratio) was titrated into a cuvette (Hellma) containing 100 nM-fluorescein-labelled peptide in 900 μL of binding buffer (20 mM-HEPES pH 7.3, 1 mM-EGTA, 2 mM-MgAc, 55 mM-KAc, 2 mM DTT, 10% glycerol, 150 mM-NaCl and 0.25 mM-GTP). In competition assays, test unlabelled peptides were titrated into a cuvette containing 100 nM-fluorescein-labelled AID-NES peptide, 7 μM-Crm1 and 7 μM-RanQ69L-GTP. Fluorescence was measured using a Cary Eclipse spectrophotometer with λ_ex_=480 nm and λ_em_=530 nm. Excitation and emission slits were 20 nM. Anisotropy was measured with a 5 s integration time. Proteins were titrated into the cuvette with a Hamilton-Microlab titrator allowing 50 s stirring after each titration step and a wait of 10 s before anisotropy was recorded. *K*_D_ values were calculated using the program Datafitter (D. Veprintsev) based on at least two independent experiments.

### Assaying CSR

pMx-GFP expressing vectors containing human AID were generated by PCR-mutagenesis and co-transfected with pEco packaging vector into 293T cells using Genejuice to generate human AID-encoding retroviruses. CSR was then measured following retroviral transduction of LPS+IL4 stimulated AID^−/−^ B cells as described 12, with animal procedures performed under UK Home Office PPL80/2226.

### Localisation

pMx-GFP expressing vectors containing HA-tagged (C-terminal) human AID were generated by PCR-mutagenesis and transfected using Genejuice. Cells were replated onto glass slides 24 h after transfection, fixed in 2% paraformaldehyde, permeabilised with 0.4% Triton X-100 and stained with Y-11 anti-HA rabbit polyclonal IgG (Santa Cruz Biotechnology), developing with AlexaFluor 568-conjugated goat anti-rabbit IgG (Invitrogen) and counterstaining with DAPI/Vectashield (Vectorshield).

### AID Abundance

B cells from AID^−/−^ mice were incubated for 24 h in the presence of LPS+IL4 prior to transduction with GFP-expressing retrovirus encoding HA-tagged human AID and culturing for 3 days as described 17. Cells (10^6^) were heated in reducing SDS-sample buffer (50 μL), subjected to SDS/PAGE and AID abundance monitored by Western blotting using biotinylated mouse anti-hAID mAb h52–1 18 developing with SA-HRP (GE Healthcare). GFP was detected using rabbit anti-GFP antiserum (Santa Cruz Biotechnology) developing with HRP-conjugated goat anti-rabbit IgG (Southern Biotech).

## Abbreviations

AID: activation-induced deaminase
CSR: class-switch recombination
NES: nuclear-export sequence
PKI: protein kinase inhibitor

## References

[1] Alt FW, Honjo T (2007). AID for Immunoglobulin Diversity, Adv. Immunol.

[2] Di Noia JM, Neuberger MS (2007). Molecular mechanisms of antibody somatic hypermutation. Annu. Rev. Biochem.

[3] Revy P, Muto T, Levy Y, Geissmann F, Plebani A, Sanal O, Catalan N (2000). Activation-induced cytidine deaminase (AID) deficiency causes the autosomal recessive form of the Hyper-IgM syndrome (HIGM2). Cell.

[4] Ta VT, Nagaoka H, Catalan N, Durandy A, Fischer A, Imai K, Nonoyama S (2003). AID mutant analyses indicate requirement for class-switch-specific cofactors. Nat. Immunol.

[5] Zhu Y, Nonoyama S, Morio T, Muramatsu M, Honjo T, Mizutani S (2003). Type two hyper-IgM syndrome caused by mutation in activation-induced cytidine deaminase. J. Med. Dent. Sci.

[6] Patenaude AM, Orthwein A, Hu Y, Campo VA, Kavli B, Buschiazzo A, Di Noia JM (2009). Active nuclear import and cytoplasmic retention of activation-induced deaminase. Nat. Struct. Mol. Biol.

[7] Brar SS, Watson M, Diaz M (2004). Activation-induced cytosine deaminase (AID) is actively exported out of the nucleus but retained by the induction of DNA breaks. J. Biol. Chem.

[8] Ito S, Nagaoka H, Shinkura R, Begum N, Muramatsu M, Nakata M, Honjo T (2004). Activation-induced cytidine deaminase shuttles between nucleus and cytoplasm like apolipoprotein B mRNA editing catalytic polypeptide 1. Proc. Natl. Acad. Sci. USA.

[9] McBride KM, Barreto V, Ramiro AR, Stavropoulos P, Nussenzweig MC (2004). Somatic hypermutation is limited by CRM1-dependent nuclear export of activation-induced deaminase. J. Exp. Med.

[10] Imai K, Zhu Y, Revy P, Morio T, Mizutani S, Fischer A, Nonoyama S (2005). Analysis of class switch recombination and somatic hypermutation in patients affected with autosomal dominant hyper-IgM syndrome type 2. Clin. Immunol.

[11] Barreto V, Reina-San-Martin B, Ramiro AR, McBride KM, Nussenzweig MC (2003). C-terminal deletion of AID uncouples class switch recombination from somatic hypermutation and gene conversion. Mol. Cell.

[12] Geisberger R, Rada C, Neuberger MS (2009). The stability of AID and its function in class-switching are critically sensitive to the identity of its nuclear-export sequence. Proc. Natl. Acad. Sci. USA.

[13] Englmeier L, Fornerod M, Bischoff FR, Petosa C, Mattaj IW, Kutay U (2001). RanBP3 influences interactions between CRM1 and its nuclear protein export substrates. EMBO Rep.

[14] Engelsma D, Bernad R, Calafat J, Fornerod M (2004). Supraphysiological nuclear export signals bind CRM1 independently of RanGTP and arrest at Nup358. EMBO J.

[15] Dong X, Biswas A, Chook YM (2009). Structural basis for assembly and disassembly of the CRM1 nuclear export complex. Nat. Struct. Mol. Biol.

[16] Askjaer P, Jensen TH, Nilsson J, Englmeier L, Kjems J (1998). The specificity of the CRM1-Rev nuclear export signal interaction is mediated by RanGTP. J. Biol. Chem.

[17] Di Noia JM, Williams GT, Chan DT, Buerstedde JM, Baldwin GS, Neuberger MS (2007). Dependence of antibody gene diversification on uracil excision. J. Exp. Med.

[18] Conticello SG, Ganesh K, Xue K, Lu M, Rada C, Neuberger MS (2008). Interaction between antibody-diversification enzyme AID and spliceosome-associated factor CTNNBL1. Mol. Cell.

